# Nutrition—sensitive agricultural interventions and maternal and child nutrition outcomes in arid and semi—arid lands of Kenya

**DOI:** 10.3389/fnut.2025.1465650

**Published:** 2025-02-18

**Authors:** Frederick K. E. Grant, Dorcas Amunga, Chalmers K. Mulwa, Mukani Moyo, Norman Kwikiriza, Jack Malit, Lucy Mwaura, Joyce Maru, Simon Heck

**Affiliations:** ^1^International Potato Center, Kampala, Uganda; ^2^International Potato Center, Nairobi, Kenya; ^3^International Potato Center, Lima, Peru

**Keywords:** humanitarian settings, arid and semi-arid lands, knowledge and practices, nutrition, Kenya

## Abstract

**Background:**

In regions facing chronic stress such as the arid and semi-arid lands (ASALs) of Kenya, there is poor quality of diet among women and children in humanitarian situations, mainly due to multiple climatic shocks that exacerbate local food systems. The objective of this study was to assess the effect of household participation in climate smart nutrition-sensitive agriculture (NSA) interventions on maternal and young child nutrition outcomes in Makueni, Garissa and Tana River counties.

**Methods:**

From March 2020 to October 2023, the International Potato Center and partners (World Food Program, Ministries of Agriculture and Health) implemented an NSA intervention in Makueni, Garissa and Tana River counties. The intervention comprised of household participating in three main activities: (1) access to orange fleshed sweetpotato (OFSP) vines; (2) participation in nutrition education activities and (3) receiving and utilizing infant feeding toolkits (Healthy Baby Toolkit/HBT). Approximately 3 months after intervention activities, we conducted a cross-sectional survey in intervention communities to assess effect of particpation in the interventions on maternal and child nutrition outcomes. The study utilized the doubly robust *inverse probability weighting regression adjustment* (IPWRA) estimator to evaluate the impact of participation in the project intervention on nutrition outcomes. Caregivers’ knowledge of nutrition, health and childcare, women (MDD-W) and young child dietary diversity (MDD-C), vitamin A (VA) intakes, minimum meal frequency (MMF), and minimum acceptable diet (MAD) for children 6–23 months were analyzed. The comparison of means and proportions was assessed using Student’s t-test and the Chi-square test, respectively, between the caregivers participating in NSA interventions and non-participants. The impact of the level of participation in NSA interventions and information on how to utilize these to improve infant and maternal feeding in the household on caregiver knowledge and practices scores was examined using regression analysis.

**Results:**

Of 494 caregivers surveyed, 72% indicated to have participated in at least one study intervention. In adjusted analyses, participation in at least one of the study interventions was significantly associated with improved caregiver nutrition [*β*: 0.943, *p* < 0.05], and VA [0.613, *p* < 0.05] knowledge scores and young child MMF [0.202, *p* < 0.05] and MAD [0.111, *p* < 0.05]. Participation in all three interventions (nutrition training, use of infant feeding toolkit and access to OFSP vines) significantly increased VA knowledge among caregivers (*p* ≤ 0.05) and infant MMF (*p* ≤ 0.05). While the use of infant feeding toolkit and access to OFSP vines alone had a significant positive effect on MDD-W and MDD-C (*p* ≤ 0.05).

**Conclusion:**

The findings show the need to integrate climate-smart NSA interventions in humanitarian settings to improve nutrition among women and young children. Such interventions can potentially build resilience among populations in these fragile environments to better withstand various shocks.

## Introduction

The path toward the attainment of the Sustainable Development Goal 2 (SDG #2) which encompasses food security and nutrition is still unclear for most low- and middle-income countries (LMICs), particularly in sub-Saharan Africa (1). The burden of maternal and child undernutrition has remained unacceptably high in majority of the LMICs (2). In Kenya, 18% of children under 5 years are stunted, with higher prevalence in rural areas (3). Adequate (meeting minimum diet diversities, meal frequencies, and minimum acceptable diets) diets can reduce and/or prevent the prevalence of various forms of malnutrition and subsequently, forms of undernutrition associated with high mortality rates in young children (4). Unfortunately, diets of vulnerable population groups such as children under 5 years and women of reproductive age (WRA) rarely meet their nutritional needs (5). Only 37% of children aged 6 to 23 months consume an adequately diverse diet while 31% are fed a minimum acceptable diet (MAD) in Kenya (3). WRA diets are equally sub-optimal (6). Diet quality among those living in ASAL are far worse due to multiple climatic shocks that exacerbate local food systems (7).

In recent years, efforts have been made to support diversification and encourage opportunities that improve access, availability, and utilization of nutritious foods (8). Nutrition-sensitive agricultural (NSA) programs can address the underlying determinants of malnutrition by enhancing food security (access, availability, and utilization of nutrient-rich foods), promoting adequate feeding (age-appropriate dietary practices), and cultivating healthy food environments (9). Well-designed and deliberately implemented NSA programs can improve household access to nutritious foods, possibly leading to improvements in diets, especially among young children (10). Evidence has also shown that food-based interventions have the potential to substantially reduce malnutrition, such as vitamin A deficiency (VAD), among vulnerable populations (11). Specifically, interventions with biofortified orange-fleshed sweetpotato (OFSP) have been shown to improve maternal and young child vitamin A (VA) status (11–15). OFSP, as a staple food, can supply significant amounts of VA and energy simultaneously, thus helping to address both VAD and undernutrition, respectively.

Considering that 70% of the rural households in Kenya are engaged in agriculture farming (16) NSA strategies such as biofortification hold promise as complementary strategies to improve the nutritional status of these households. Unfortunately, the current (8) body of evidence on the contribution of nutrition-sensitive agriculture interventions to improving diet quality among young children and WRA in ASAL communities is limited. In addition, while nutrition-sensitive programs have been shown to improve maternal and child diet quality when they include strong behavior change communication (BCC) interventions (10, 17, 18), more research is needed to understand their contribution in the context of ASALs. In this study, a multi-country nutrition-sensitive agriculture program known as the Development and Delivery of Biofortified Foods at Scale (DDBIO) project was implemented by the International Potato Centre (CIP) and its partners over a period of 3 years (11, 19). In Kenya, the program targeted vulnerable communities living in ASALs and was implemented as a package that combined three main interventions: the provision of OFSP vines to agro-pastoral households; agricultural and nutrition education, training, and support (focusing on OFSP production and maternal and child nutrition) as well as the distribution of child feeding bowls (Healthy Baby Toolkit) to promote optimal complementary feeding (20). The objective of this current study was to assess the effect of participation in nutrition-sensitive agriculture interventions implemented within the DDBIO project on maternal and young child nutrition outcomes among participating ASAL communities in Kenya.

## Methods

### Study area

This post-intervention survey was conducted in three intervention counties where the DDBIO project was jointly implemented by CIP, County Governments, and the World Food Program (WFP) in Kenya: Makueni, Garissa, and Tana River counties. Poor nutrition practices and low household income are a common characteristic in these counties; however, each county has the potential for integrating OFSP into their farming systems and expanding its production. The project intervention counties were purposively selected because they fall within areas of intervention by WFP Kenya besides having the potential to build resilience of the communities (especially, Garissa and Tana River counties) to sustainably produce own food crops and wean vulnerable households from rationings from WFP. The villages in the project intervention counties were enumerated in January 2023 in preparation for sampling the villages and households to be surveyed. Garissa County experiences an average annual rainfall of 250 – 350 mm and the crops widely farmed are maize, rice, green gram, cowpea, and banana (21). Makueni County receives an average annual rainfall of 300–1,200 mm (22). Farming is the main economic activity in the County (Makueni) with crops widely produced including green gram, sorghum, maize, mango, cowpea, bean, pigeon pea and citrus fruits (22). Tana River County has a relatively dry and hot climate throughout the year, receiving less than 500 mm of precipitation per year in most areas, and the rest less than 1,000 mm per year (23). Livestock rearing, through pastoralism, is the main economic activity, however, crop production (mangoes, pawpaw, citrus, bananas, and melons) is also practiced but mainly along the Tana River (23). Bimodal rainfall patterns received in these three counties are generally unreliable with long lean (little rain) seasons in between experienced due to climatic shocks and contributing to low agricultural productivity and livelihoods. Biofortified varieties of sweetpotato were introduced in these counties as they are resilient (short maturity period, drought tolerant, high productivity per unit area) and nutritious, therefore suitable for such agroecology. In these communities, sweetpotato is produced mainly for home consumption and is consumed as boiled, roasted, or incorporated with other commonly consumed foods as a mixed dish. To document effect of the DDBIO project intervention on maternal and young child nutrition outcomes, we conducted a cross-sectional survey from January to February 2023 that captured household socio-demographics, child caregiving and dietary practices, and crop production and utilization. The survey was conducted among 636 households in intervention communities with a pregnant or lactating woman or a child less than 5 years old.

### Study intervention

The study implemented three types of interventions: (1) purchase of OFSP vine; (2) Nutrition education and (3) Healthy Baby Toolkit/HBT. The details of these interventions are described below:

OFSP vine purchase: Farming households using either rain-fed or irrigation agriculture in the counties of focus were selected by Ward Agriculture Extension Officers (WAEOs). The households received training on good agriculture practices, sensitized on OFSP production and linked to decentralized vine multipliers (DVMs) where they purchased OFSP vines at a subsidized rate for multiplication at their households. Farmers received extension services from WAEOs routinely during the planting and harvesting season. OFSP demonstration plots were also established in four health centers in Makueni for learning, seed bulking and subsequent distribution to farming households.

Nutrition Education and innovative feeding toolkits: The caregivers participated in monthly nutrition meetings led by community health volunteers (CHVs) that dialogued on nutrition for optimum health for infants, young children, adolescents, and pregnant and lactating women. At each monthly group meeting, the CHVs used desk-sized dialog cards containing various lessons to provide improved maternal, infant, and young child nutrition counseling (24). The main lessons were on: (1) nutrition during pregnancy and breastfeeding; (2) infant and young child (complementary) feeding; (3) food preparation (4); hygiene and sanitation; (5) food production through creating kitchen gardens. These messages were similar for the nutrition group meetings in all the three counties. However, in Makueni county, the nutrition training curriculum also included: (1) benefits of OFSP; (2) growing OFSP; (3) vitamin A and (4) biofortification. During these meetings, CHVs led the group members in cooking demonstrations that utilized OFSP and other locally available nutritious and VA-rich foods.

Further, innovative feeding toolkits – healthy baby toolkits/HBTs - for feeding young children, were distributed to each caregiver. They were then taken through training on how to correctly use the HBT in feeding young children. The training centered around how to use the HBT in measuring age-appropriate amount of food, determining the right food texture, and how often (frequency) to feed children under 2 years. The innovative feeding toolkit (also referred to as the Healthy Baby Toolkit/HBT), designed for use by infants 6–23 months of age, consists of a bowl with lines and symbols that cue age appropriate meal frequency and volume, a slotted spoon that promotes optimal thickness of infant foods and a pictorial counseling card that uses locally adapted images to convey use of the toolkit to achieve optimal infant and young child feeding practices as well as messages on dietary diversity, handwashing and safe preparation of food and water (20, 25, 26).

### Study population

The study population was households involved in agricultural activities who either have at least one child 6–59 months or a woman 17–45 years of age.

### Inclusion criteria

Eligible participants were women aged 17–45 years of age, residing in the study villages and who are either the biological mother or primary caretaker of a child (6–59 months) or who have a confirmed pregnancy. Finally, women must have resided in the study villages during the period of intervention from September 2019 to December 2022.

### Exclusion criteria

Ineligible participants included women younger than 17 or older than 45 years of age who are not the primary caretaker of a child (6–59 months of age). Women who did not reside full-time in the study villages during the period of the intervention were not eligible.

### Ethical clearance

This study was conducted following the guidelines laid down in the Declaration of Helsinki. The National Commission for Science, Technology and Innovation/NACOSTI, Kenya, approved all procedures involving research study participants. Additional approval was obtained from the research ethic committee of the International Livestock Research Institute’s Institutional Research Ethic Committee (ILRI - IREC) in Nairobi, Kenya. Written informed consent was obtained from all study participants. No known risks were presented to the study population beyond some possible discomfort due to the need to assess certain targeted behaviors of the intervention (OFSP knowledge, production, diet practices, food consumption patterns, etc.) or inevitable survey procedures.

### Estimation of sample size

Using standard sampling techniques (27) with unknown population of interest, the minimum sample to allow for sufficient analytical power to detect differences in outcomes between the households within different levels of participation is 385 households, i.e.,


$$n=\left(\frac{Z^{2}PQ}{d^{2}}\right)$$

Where n is the sample size and P is the proportion of the population of interest, set at 0.5 which statistically results in a sufficient and reliable size when the population proportion is unknown with certainty; d is the significance level set at 5% to remove 95% bias in sampling, resulting to Z value of 1.96; Q is the weighting variable computed as 1−P. Plugging these values in above equation results to a sample size of 385 observations.

Given the proposed impact evaluation methods (discussed hereafter) that rely on similarities between treatment and control units, with the possibility of discarding units with non-conforming characteristics, this sample was increased to 600 households. The sample size of the survey was based on the principle to enable a comprehensive and objective comparison of the main knowledge and practices (KP) outcome of household weekly frequency of OFSP consumption among project participants. This outcome assumes an improvement in the proportion of households consuming OFSP at least once a week from 0.5% pre-DDBIO interventions to 10% post-interventions. This assumption was based on findings from our previous work in Western Kenya (15) with the estimated sample size allowing for comparison between pre- and post-intervention proportions and considering the complex survey design effect of 1.5 (28). To cater for a 15% potential non-response, the sample size was increased to 636 households. The sample size was proportionately distributed among the three counties using the probability proportional to the size sampling technique (29). The sample size would allow comparisons for OFSP knowledge, growing practices and consumption, and dietary practices among households between the various intervention arms.

### Sampling procedure

The study employed a multistage sampling procedure to select the study respondents. In the first stage, project intervention sub-counties, wards and villages in the three selected counties were listed with help of local WFP staff and project implementation partners, and probability proportional to size (PPS) sampling used to select 417 beneficiary households in wards where the project was implemented. A similar strategy was employed to select 219 non-beneficiary households in wards where the project was not implemented to form a comparison group in the impact analyses strategy discussed hereafter. This sampling strategy resulted in a total of 417 and 219 project beneficiary and non-beneficiary households respectively, spread out across the 35 villages in the study area. This resulted in a total of 636 participants from across these administrative units.

During enumeration, 25 households with children between 6 and 59 months old were selected in each village by the CHVs. Among these households, a maximum of 18 were randomly selected for individual interviews. All eligible respondents provided informed consent before being interviewed. Therefore, 630 households, who were represented by the primary caregiver of a young child, were identified for enumeration as part of the study. The study was oversampled (from 600 to 636) to ensure we have enough power to be able to detect potential differences in other outcomes.

### Interview modules

Trained enumerators used a standardized, structured tablet-based questionnaire to directly record the responses from each respondent. The survey tools were developed by the DDBIO staff in collaboration with project implementing partners (IPs). The tools were then reviewed for completeness and accuracy and pre-tested before administering in the field for data collection. In consultation with the IPs, the questionnaire was modified and finalized based on the results from the pre-test. The questionnaire was divided into modules, with questions in each module intended to capture various information, knowledge, and practices among target population about sweetpotato in general and OFSP in particular (17). The modules were as follows:

- Module A: Household Contact Information.
- Module B: Household Characteristics. The characteristics of households (number of members and assets), household head (age, education, and employment), mother (age, relationship to household head, marital status, education, employment, and parity), and children (age and sex).
- Module C: Dietary Diversity. Dietary diversity of the household and caregivers utilized a questionnaire combining the Helen Keller International (HKI) food frequency module informing on the frequency of VA-rich food consumption (30) and the WHO 24-h recall method that focuses on the dietary diversity and acceptable diet (31).
- Module D: Household Food Security. Household food security was assessed using the FANTA Household Food Insecurity Assessment Scale (HFIAS), which has been previously validated in this context (30).
- Module D: Health and Nutrition knowledge and practices (KPs). Sought the mothers’ or caregivers’ knowledge of nutrition and VA, including OFSP and other VA-rich foods.
- Module E: Agriculture. Sought information on agricultural production, use of agricultural products, and income derived from agriculture, including OFSP and knowledge about sweetpotato agronomy.
- Module F: Project Exposure and Uptake: That included access to OFSP vines for planting; attending OFSP field days and demonstrations; and if ever participated in pregnant and breastfeeding mother’s club run by the village based CHVs, if ever received and utilized a healthy baby toolkit.
- Module F: Trends in sweetpotato production.

During enrolment for each respondent, data were collected on basic socio-demographic characteristics, such as age, marital status, education, occupation, household size, and composition. Data on agricultural resources and household assets were also collected to provide a context for understanding the overall results of the study.

### Field methods for data collection

The survey had a team of fieldworkers for data collection comprising of 10 enumerators, a team leader among enumerators, and a CIP staff as a supervisor. The team leader had the responsibility for visiting teams in the field, ensuring that households are selected accurately, and adequate survey tools and other logistics are available. The supervisor was also responsible for deciding how to overcome unexpected problems. Each problem encountered and each decision made were recorded and included in the supervision report. At the end of each workday, the team leaders conducted a wrap-up session with the team to discuss any problems encountered during the day and reviewed all questionnaires and tracking forms to ensure accuracy and completeness. After a review of each completed computer-assisted personal interview (CAPI)-entered data, a backup was created before closing the day’s work through Bluetooth technology.

The interview of each caretaker of the eligible and selected household (HH) took approximately 50–70 min, and questions were asked in the Kiswahili language. Each interview was conducted at the home of the participant after she was presented with an informed consent. At the end of each day, the team leader with the assistance of the supervisor reviewed the completed CAPI questionnaires and discussed issues and concerns about the day’s interviews. The issues were addressed using field notes, and if necessary, interviewers would return to pertinent HHs to correct the errors.

### Variable specifications

#### Dependent variables

Eight outcome variables that describes maternal knowledge and practices as well as young child dietary intakes were constructed. The variables were: (1) VA knowledge score (VAKS); (2) nutrition knowledge score (NKS); (3) dietary diversity score for women; (4) caregiver VA intake; (5) young child VA intake; (6) Minimum dietary diversity for children; (7) Minimum meal frequency; and (8) Minimum acceptable diet.

VAKS and NKS were constructed from key variables using equally weighted summative item score (17). The selected items for knowledge scores were equally important and homogenous hence, weights were not applied in generating these scores (32). The VAKS ranged from 0 to 5 points, while the NKS ranged from 0 to 24 points.

The primary caregiver health and nutrition practices of interest were dietary diversity score (DDS) score for both caregiver/women (MDD-W) and young children (MDD-C), as well as minimum meal frequency (MMF) and minimum acceptable diet (MAD) for children 6–23 months. The DDS were derived from a 24-h food recall, adding the number of different food groups out of 10 for MDD-W and out of 8 for MDD-C, which were consumed by the caregiver or child within the past 24 h (33–35), p. 360. Specifically, for MDD-W, 10 food groups were included in the index calculation for households: (1) Grains, white roots and tubers, and plantains, (2) Pulses (beans, peas and lentils), (3) Nuts and seeds, (4) Dairy, (5) Meat, poultry and fish, (6) eggs, (7) Dark green leafy vegetables, (8) Other vitamin A-rich fruits and vegetables, (9) Other vegetables, (10) Other fruits. The OFSP was categorized as other vitamin A rich fruits and vegetables. Each food group was scored as 0 if not consumed during the past 24 h and 1 if consumed during the same period. The dietary diversity index was obtained by summing the scores for the 10 food groups. Therefore, the possible range of the dietary diversity index was from 0 to 10. Women with dietary diversity scores ≥5 were classified as having met the minimum dietary diversity, whereas those with scores <5 were classified as not meeting MDD. For computation of a young child’s (aged 6–59 months) DDS (MDD-C), the food groups used were as follows: (1) breast milk; (2) grains, roots, and tubers; (3) legumes and nuts; (4) dairy products; (5) flesh foods (meats/fish/poultry); (6) eggs; (7) vitamin A-rich fruits and vegetables; and (8) other fruits and vegetables. Each food group was scored as 0 if not consumed during the past 24 h and 1 if consumed in the same period. The dietary diversity index was obtained by summing up the scores for the eight food groups. The possible range of the dietary diversity index was from 0 to 8. Children with dietary diversity scores ≥5 were classified as meeting the minimum dietary diversity, whereas those with scores <5 were classified as not meeting MDD.

The frequency of VA consumption score was calculated using the HKI food frequency index model to assess the household risk level of VAD (30). This model counts the frequency of how certain foods are eaten over time although it suffers from a failure to capture actual amounts of each food consumed. However, this model was validated against biochemical indicators and can be used to adequately predict whether VAD is a public health problem in the population. A household was at risk of VAD if the mean frequency of consuming VA from animal sources was 4 days per week or less or the mean frequency of total consumption of animal and plant sources of VA was 6 days per week or less. The frequency of the VA consumption score was calculated by first summing the number of days during the previous week the child or the caregiver consumed VA-rich food from an animal source. Then, the number of days the child or caregiver consumed VA-rich food from a plant source was summed and divided by 6. The following formula was used in calculating the index: Weighted total consumption days (Cw) = Total number of days animal sources of Vitamin A consumed (TVA) + Total number of days plant sources of Vitamin A consumed (TAP) divided by 6.

The weighted VA consumption score (C) is equal to the total number of days the child or mother consumed VA-rich food items from animal sources plus the adjusted consumption from the plant sources. The following animal and plant sources were included in the estimation of the index.

- Animal sources: eggs with yolk, fresh silverfish (*omena*) with intact liver or dried silverfish (*omena*) with intact liver, liver from any animal or bird (e.g., chicken) or fish, butter, cod liver oil, VA-fortified margarine (Blue Band) or fortified oil, Cerelac (fortified packaged cereal), infant formula (e.g., NAN, etc.), blood added as an ingredient (Mutura), and VA-fortified sugar.
- Plant sources: sweetpotato leaves, all kinds of dark green vegetables, carrots, ripe mango, pumpkin, ripe papaya, and orange- and yellow-fleshed sweetpotato.

The cut-off point for adequate frequency of VA intake was 6 for the weighted consumption score.

The minimum meal frequency (MMF) serves as a proxy for a child’s energy requirements. It examines the number of times a child aged 6–23 months received foods other than breastmilk within a 24-h period. The minimum number is specific to the age and breastfeeding status of the child The (MMF) for children 6–23 months was estimated as follows: twice for breastfed infants aged 6–8 months, three times for breastfed children aged 9–23 months, and four times for non-breastfed children aged 6–23 months (36). The minimum acceptable diet (MAD) is a binary indicator of infant and young child feeding practice that assesses the quality and sufficiency of a child’s diet between the ages of 6–23 months (36, 37). It is a composite indicator derived from MDD and MMF. MAD for currently breastfeeding children is defined as “receiving at least the MDD and MMF for their age during the previous day.” Similarly, MAD for children not currently breastfeeding is defined as “receiving at least the MDD and MMF for their age during the previous day as well as at least two milk feeds” (36, 37).

### Independent variables

The primary independent variable for this study was participation in at least one out of three of the project’s nutrition-sensitive agriculture interventions: receiving OFSP vines, receiving innovative infant feeding tools (healthy baby toolkit/HBT), or participating in nutrition group meetings. Based on review of the literature and our previous studies in Kenya and Tanzania (17, 38), we hypothesized *a priori* that household participation in any of the interventions was a key source of nutrition and health knowledge acquisition and thus, practices. Further, we identified other maternal and household level factors as potential confounders in the association between exposure to any of the above project interventions and nutrition and health knowledge and practices.

Primary caregiver socio-demographic characteristics, such as age, marital status, educational status, involvement in agricultural activity and selling agriculture products, engagement in salaried employment, cultivation, and consumption of sweetpotato (OFSP), were identified as potential confounders in the association between behavior change and KP outcome variables.

At the household level, we considered the status and educational level of the household head as potential confounders of caregiver’s participation in any of the project’s interventions.

We used the household wealth index as a proxy for the socioeconomic status of the household. This was constructed by summing the values of different predominantly discrete data household variables, such as the type of housing and roofing, the presence and type of toilet, the source of water during the dry season, and the source of cooking fuel, as well as the possession of durable household assets such as radio, television, telephone/mobile, solar panels, gas cooker, bicycle, water pump, motorcycle, car /truck, tractor, and generator. A wealth index based on ordinal variables for these data was created to allow comparison across sites (39).

### Data management and analyses

#### Data management

CSPro-supported CAPI data entry system was employed to collect and collate data. In CAPI, the enumerators used smartphones to enter responses on site during the interview. The CAPI application enabled interviews to be conducted face-to-face and determined the question order and performed editing of responses as well as skip patterns. CAPI, therefore, offered a flexible approach to collecting and editing the data, resulted in better data quality, and improved the efficiency of interviewing and final data processing.

Efforts were made to ensure consistency in the survey execution at every household. All the data were subsequently combined for all the sampled villages and households through a centralized database management system. After data collection and collation in CsPRO, Stata version 15 (StataCorp., College Station, TX) was used to generate reports for missing data checks, range, and other basic logics.

### Data analyses

In the absence of a baseline and randomization into treatment, the study relied on quasi-experimental impact evaluation methods to analyze the data. The approach involves mimicking a randomized control trial (RCT) to compare outcomes of interest for the treated households with those of non-treated households to elicit treatment impact. To reduce bias, households with similar pre-existing characteristics, such as socioeconomic conditions, are compared and the differences average to estimate treatment impact. Specifically, this study utilized the doubly robust *inverse probability weighting regression adjustment* (IPWRA) estimator to evaluate impact of participation in the project intervention on nutrition outcomes. The approach combines an outcome regression with a model for exposure to treatment, based on propensity score methods, in estimating the effect of the treatment exposure on the outcome (40–42). Following (43), we first estimate the propensity score, p(w), for the probability of being exposed to intervention treatments based on *X*;


$$p\left(x\right)=P\left(t=1/X\right)$$

Next, we use linear outcome functions to estimate *α* and *β* parameters using inverse probability weighted least squares as below:


minα1,β1∑i=1Nyi−α1−β1XipXiϒ^ift=1

minα0,β0∑i=1Nyi−α0−β0Xi1−pXiϒ^ift=0

The average treatment effect is then obtained as,


$$\text{ATE}T_{\text{IPRWA}}=N_{T}^{-1}\sum_{i=1}^{N_{T}}\left[\left({\hat{\alpha }}_{1}-{\hat{\beta }}_{1}X_{i}\right)-\left({\hat{\alpha }}_{0}-{\hat{\beta }}_{0}X_{i}\right)\right]$$

where ${\hat{\alpha }}_{1},{\hat{\beta }}_{1}$ and ${\hat{\alpha }}_{0},{\hat{\beta }}_{0}$ are the estimated inverse probability weighted parameters for the treatment and non-treated group, respectively; $N_{T}$ is the number of treated observations in our sample.

After data cleaning, descriptive statistics such as proportions and frequencies for categorical variables and mean with standard errors for continuous variables were generated. We compared socio-demographic characteristics of study participants between the two groups (those receiving at least one of the three project interventions vs. none) by using Chi-square and Fisher’s exact tests for frequencies, and Student’s *t*-test for continuous variables in bivariate unadjusted analyses. We compared the differences in caregiver nutrition and VA knowledge and dietary diversity and VA intake for both caregivers and children, as well as minimum meal frequency and minimum acceptable diet for young children between the groups.

We examined the differences between participants/treated and non-participants/non-treated in project interventions using cluster-adjusted regression analysis accounting for cluster sampling and the hierarchical nature of the data. Multiple linear regression was used to assess the impact of project intervention activities (none, 1, and > 1 of the interventions) on caregiver nutrition knowledge and improved caregiver and young child dietary diversity, minimum meal frequency and minimum acceptable diet (for children 6–23 months). We adjusted for the following caregiver and household-level potential confounding factors: maternal age, education, marital status, salaried employment, gender of household head, size of household, wealth index, distance to source of water, cultivation of sweetpotato, and household total land area for farming. We employed the stepwise backward elimination model, removing covariates with the largest *p*-value at each step until the remaining variables were significant at least *p* = 0.05 in the final model. Data are shown as mean ± standard errors (SEs). For all analyses, a *p*-value of 0.05 was deemed statistically significant.

## Results

The socio-demographic characteristics of the study participants and households, together with their respective participation levels are presented in Table 1. A total of 636 households were surveyed in the study target areas of Garissa, Tana River, and Makueni counties. Of these, 494 were primary caregivers of children under 5 years (0–59 months) of age out of which 260 were aged between 6 and 23 months. In this study, the reference child was a child aged between 6 and 23 months of which infant nutrition outcomes (minimum diet diversity for children or MDD-C, minimum meal frequency or MMF, and minimum acceptable diet - MAD) were reported, having in mind these young child dietary practices are only applicable to this age group: 6–23 months old. Overall, 356 (72%) of caregivers indicated to have participated in at least one study intervention and either purchased planting materials of vitamin A biofortified orange-fleshed sweetpotato (OFSP) for cultivation, or attended nutrition group meetings and received information on nutrition social behavior change communication (SBCC), or received innovative feeding toolkits (healthy baby toolkit/HBT) and information on how to utilize these to improve infant and maternal feeding in the household. Of these caregivers who participated in any of the study interventions, 50% (*n* = 178) were involved in only one of the interventions (described previously) with the other 50% participating in more than one of these interventions. A total of 138 (28%) of the surveyed caregivers did not participate in any of the study’s interventions. The mean age of participating caregivers was 33 years with no significant differences between the age of the caregivers participating or not participating in the study’s interventions. The majority of the caregivers were married (82%) and this did not differ by level of participation in the study’s intervention. However, a significantly greater proportion of women who participated in the study’s interventions had either completed primary school (43% vs. 37%) or higher education (56% vs. 46%) than non-participants (*p* ≤ 0.05). Women who participated in more than one intervention were more likely to cultivate OFSP (57% vs. 47; *p* = 0.088) and to be from households in the highest tertile of the wealth index (40% vs. 27%; *p* ≤ 0.05) compared to those who did not participate in any of the interventions. About 96% of caregivers from households participating in more than one intervention had access to their own toilet facilities compared to only 80% of non-participants.

**Table 1:** Participant and household demographics by whole sample and level of participation.

Variable	Overall	Caregiver participation in study interventions			
		None (*n* = 138)	One (*n* = 178)	>1 (*n* = 178)	*p*- value
Age of caregiver, years	32.8 ± 11.0	33.2 ± 12.5	33.4 ± 10.9	31.9 ± 9.7	0.409
Marital status of caregiver					
Single	91 (18.4)	28 (20.3)	33 (18.5)	30 (16.9)	0.736
Married	403 (81.6)	110 (79.7)	145 (81.5)	148 (83.1)	
Caregiver education level					
None	39 (7.9)	24 (17.4)	13 (7.3)	2 (1.1)	0.000**
Primary school level	203 (41.1)	51 (37.0)	76 (42.7)	76 (42.7)	
Above primary school level	252 (51.0)	63 (45.6)	89 (50.0)	100 (56.2)	
Household size	5.2 ± 2.1	5.5 ± 2.3	5.2 ± 2.0	5.0 ± 1.9	0.047**
Gender of household head (%)					
Male headed	537 (84.4)	177 (80.8)	192 (87.3)	168 (85.3)	0.163
Female headed	99 (15.6)	42 (19.2)	28 (12.7)	29 (14.7)	
Household head education level					
None	70 (11.0)	47 (21.5)	17 (7.7)	6 (3.1)	0.000**
Primary school level	264 (41.5)	87 (39.7)	93 (42.3)	84 (42.6)	
Above primary school level	302 (47.5)	85 (38.8)	110 (50.0)	107 (54.3)	
Young child (6–23 months) OFSP consumption within the past 24 h (%)	18 (6.9)	5 (5.4)	6 (6.9)	7 (8.6)	0.709
OFSP source (%)					
Own Farm	12 (66.7)	3 (60.0)	3 (50.0)	6 (85.7)	0.369
Markets	6 (33.3)	2 (40.0)	3 (50.0)	1 (14.3)	
Grow OFSP currently (%)	312 (50.2)	102 (46.8)	100 (47.6)	110 (56.7)	0.088**
Agricultural activity (%)					
Principal	470 (73.90)	167 (76.3)	153 (69.6)	150 (76.1)	0.191
Secondary	166 (26.10)	52 (23.7)	67 (30.4)	47 (23.9)	
Sold agriculture or livestock products (%)	176 (27.7)	62 (28.3)	66 (30.0)	48 (24.4)	0.424
Taken salaried employment (%)	115 (18.1)	32 (14.6)	51 (23.2)	32 (16.2)	0.048**
Took casual labor (%)	195 (30.7)	61 (27.9)	73 (33.2)	61 (31.0)	0.478
Been involved in informal business (%)	103 (16.2)	33 (15.1)	42 (19.1)	28 (14.2)	0.344
Been in self-employment (%)	157 (24.7)	52 (23.7)	56 (25.5)	49 (24.9)	0.915
Wealth index of household	2.00 ± 0.82	1.83 ± 0.83	1.99 ± 0.83	2.19 ± 0.75	0.000**
First tertile (Lowest)	213 (33.5)	96 (43.8)	77 (35.0)	40 (20.3)	0.000**
Second tertile (Medium)	211 (33.2)	64 (29.2)	68 (30.9)	79 (40.1)	
Third tertile (Highest)	212 (33.3)	59 (26.9)	75 (34.1)	78 (39.6)	
WASH Indicators					
Distance to the main source of water (dry)	28.42 ± 38.04	26.63 ± 32.26	27.60 ± 38.43	31.34 ± 38.04	0.384
Toilet ownership, yes	564 (88.7)	176 (80.4)	199 (90.5)	189 (96.0)	0.000**
Toilet type					
Pit latrine	528 (93.6)	164 (93.2)	183 (92.0)	181 (95.8)	0.296
Flush toilet	36 (6.4)	12 (6.8)	16 (8.0)	8 (4.2)	

^1^Mean ± Standard Deviations and *n* (%) for all such values, **Represents *p* ≤ 0.05.

Table 2 presents comparison of knowledge and practices of primary caregivers of children under 5 years of age between participants and non-participants in the study interventions. Overall, 47% of caregivers exhibited high knowledge on vitamin A; however, vitamin A knowledge scores differed significantly between participants and non-participants (59% vs. 32%; *p* ≤ 0.05). Also, there was greater likelihood of participating caregivers having higher nutrition knowledge (42% vs. 25; *p* ≤ 0.05) compared to non-participants. Women who participated in at least one of the study interventions had higher number of days of dietary vitamin A intake (3.80 vs. 3.2 days; *p* ≤ 0.05) and met the minimum dietary diversity for women (60% vs. 43%; *p* < 0.05) compared to non-participants.

**Table 2:** Knowledge and practices of primary caregivers of children under 5 years in ASALs of Kenya.

Variable		Caregiver participation in study interventions			*p*- value
	Overall	None	One	>1	
*n* (%)	494 (100.0)	138 (27.9)	178 (36.0)	178 (36.0)	
Vitamin A knowledge score^a^	3.0 ± 1.6	2.4 ± 1.8	2.9 ± 1.6	3.5 ± 1.4	0.000**
Low Score (<4)	236 (53.4)	72 (67.9)	94 (56.6)	70 (41.2)	0.000**
High Score, ref. (≥ 4)	206 (46.6)	34 (32.1)	72 (43.4)	100 (58.8)	
Nutrition Knowledge Score	11.4 ± 3.8	10.3 ± 4.0	11.6 ± 3.7	12.1 ± 3.6	0.000**
Low (<8)	79 (16.0)	33 (23.9)	24 (13.5)	22 (12.4)	0.005**
Moderate (8–13)	242 (49.0.)	71 (51.5)	89 (50.0)	82 (46.0)	
High, ref. (14–24)	173 (35.0)	34 (24.6)	65 (36.5)	74 (41.6)	
Caregiver, number of days consumed vitamin A	3.57 ± 1.62	3.21 ± 1.43	3.80 ± 1.69	3.74 ± 1.70	0.0276**
< 6 days	239 (91.9)	90 (97.8)	79 (90.8)	70 (86.4)	0.021**
≥ 6 days, ref	21 (8.1)	2 (2.2)	8 (9.2)	11 (13.6)	
Minimum diversity scores for women	4.0 ± 2.5	3.8 ± 2.6	3.9 ± 2.6	4.4 ± 2.4	0.066
Consuming ≥5 food groups	257 (52.0)	59 (42.7)	91 (51.1)	107 (60.1)	0.009**

^1^Mean ± Standard Deviations and *n* (%) for all such values, **Represents *p* ≤ 0.05, ^a^Missing values for Vitamin A Knowledge Score, *n* = 52.

In unadjusted analyses, children of participating caregivers were more likely to have improved minimum diet diversity (4.5 vs. 3.5; *p* ≤ 0.05), minimum meal frequency (2.8 vs. 2.1; *p* ≤ 0.05) and minimum acceptable diet (37% vs. 14%; *p* ≤ 0.05) compared to children of non-participating caregivers (Table 3). Such children also had greater number of days of dietary vitamin A intake (3.7 vs. 2.9 days; *p* ≤ 0.05).

**Table 3:** Diet diversity, meal frequency, minimum acceptable diet and VA intake among children between 6–23 months in ASALs of Kenya^1,2^.

Variables		Caregiver participation in study interventions			*p*- value
	Overall	None	One	>1	
*n* (%)	260 (100)	92 (35.4)	87 (33.5)	81 (31.1)	
Minimum Dietary Diversity (MDD-C)	3.81 ± 2.20	3.45 ± 2.38	3.54 ± 2.20	4.53 ± 1.81	0.001**
Meeting MDD-C	126 (48.5)	42 (45.7)	37 (42.5)	47 (58.0)	0.106
Minimum Meal Frequency (MMF)	2.40 ± 1.31	2.08 ± 1.15	2.45 ± 1.43	2.76 ± 1.27	0.002**
Meeting MMF	129 (49.6)	32 (34.8)	43 (49.4)	54 (66.7)	0.000**
Minimum Acceptable Diet	57 (21.9)	13 (14.1)	14 (16.1)	30 (37.0)	0.000**
Young Child VA Intake	3.39 ± 1.77	2.94 ± 1.53	2.94 ± 1.53	3.66 ± 1.84	0.010**
<6 days	237 (91.1)	88 (95.6)	80 (91.9)	69 (85.2)	0.051**
≥6 days	23 (8.9)	4 (4.4)	7 (8.1)	12 (14.8)	

^1^Mean ± Standard Deviations and *n* (%) for all such values, ^2^Number of caregiver vitamin A Intake = 260 since it refers to the number of caregivers of children aged between 6 to 23 months, **Represents *p* ≤ 0.05.

Table 4 presents average treatment effects on nutritional outcomes. Independently, all three interventions (nutrition training, use of infant feeding toolkit and purchase of OFSP vines) significantly increased VA knowledge among caregivers (*p ≤ 0.05*). On the other hand, nutrition training and infant feeding toolkit had a significant positive effect on caregiver VA knowledge (*p ≤ 0.05*). There was no positive effect on caregiver or child VA intake. Among the caregivers, provision of the infant feeding toolkit and OFSP vines had a significant positive effect on MDD-W (*p ≤ 0.05*). On child feeding practices, participation in all three interventions (independently) had a positive effect on children achieving the MMF (*p* ≤ 0.05), while provision of infant feeding toolkit and OFSP vines had a positive effect on MDD among children (*p* ≤ 0.05).

**Table 4:** Effect of nutrition interventions on caregiver knowledge and practices in ASALs of Kenya.

Variable	Definition	Coefficient	95% C.I	
VA knowledge	Nutrition training vs. none	0.524**	0.287	0.761
	Infant feeding toolkit vs. none	0.207**	0.207	0.100
	OFSP vines purchase vs. none	0.375	0.191	0.559
Nutrition knowledge	Nutrition training vs. none	0.090**	0.013	0.168
	Infant feeding toolkit vs. none	0.114**	0.027	0.201
	OFSP vines purchase vs. none	0.039	−0.036	0.113
Caregiver VA intake	Nutrition training vs. none	0.085	−0.031	0.201
	Infant feeding toolkit vs. none	0.094	−0.029	0.217
	OFSP vines purchase vs. none	−0.083	−0.209	0.044
Young child VA intake	Nutrition training vs. none	0.110	−0.023	0.243
	Infant feeding toolkit vs. none	0.155**	0.001	0.309
	OFSP vines purchase vs. none	−0.046	−0.179	0.087
MDD-W	Nutrition training vs. none	0.030	−0.130	0.190
	Infant feeding toolkit vs. none	0.458**	0.240	0.676
	OFSP vines purchase vs. none	0.442**	0.282	0.601
MMF	Nutrition training vs. none	0.500**	0.133	0.866
	Infant feeding toolkit vs. none	0.763**	0.303	1.224
	OFSP vines purchase vs. none	0.316**	−0.019	0.651
MDD-C	Nutrition training vs. none	−0.009	−0.147	0.130
	Infant feeding toolkit vs. none	0.399**	0.205	0.593
	OFSP vines purchase vs. none	0.392**	0.240	0.544
MAD	Nutrition training vs. none	0.418	−0.228	1.064
	Infant feeding toolkit vs. none	1.494**	0.290	2.697
	OFSP vines purchase vs. none	1.293**	0.242	2.344

**Represents *p* ≤ 0.05, Training (*n* = 281), HBT (*n* = 291), Vines (*n* = 89). Control variables: gender of the household head, age, education level, household size, number of children in the household, age of the child, access to off-farm income, market participation and distance to the source of water during dry season.

In adjusted analyses, participation in at least one of the study interventions was significantly associated with caregiver nutrition and VA knowledge scores, young child MMF and MAD, controlling for age of the caregiver, education level of the caregiver, marital status of the household head, gender of the household head, household size, market participation, access to off farm income, distance to market, currently growing sweet potato, agriculture being a principal activity, engaged in informal business, casual labor, salaried employment and wealth index (Table 5). Compared with non-participation in any of the study interventions, participation in at least one intervention was positively associated with improved caregiver nutrition [*β*: 0.943, *p* ≤ 0.05], and VA [0.613, *p* ≤ 0.05] knowledge scores and young child minimum meal frequency [0.202, *p* ≤ 0.05] and minimum acceptable diet [0.111, *p* ≤ 0.05]. In unadjusted and adjusted analyses, participation in at least one of the study’s interventions was not significantly associated with minimum diet diversity for women (MDD-W) and children (MDD-C) as well as for caregiver and young child dietary VA intakes. However, households that were cultivating OFSP at the time of the survey had improved MDD-W, while households within the highest wealth index were also likely to have improved young child VA intake. Finally, households that participated in market activities had improved MDD-W and MDD-C.

**Table 5:** Factors affecting knowledge and practices among caregivers in Makueni, Garissa and Tana River Counties, Kenya.

Outcomes	Nutrition knowledge		Caregiver VA intake		Child VA intake		VA Knowledge		MMF		MDD-W		MDD-C		MAD	
	Unadjuste β (SE)	Adj. β (SE)	Unadj. β (SE)	Adj. Β (SE)	Unadj. β (SE)	Adj. β (SE)	Unadj. β (SE)	Adj. β (SE)	Unadj. β (SE)	Adj. β (SE)	Unadj. β (SE)	Adj. *β* (SE)	Unadj. β (SE)	Adj. β (SE)	Unadj. *Β* (SE)	Adj. Β (SE)
Intercept	4.691 (1.481)		2.296 (0.868)		1.679 (0.977)		0.066 (0.745)		−0.208 (0.296)		3.565** (1.029)		1.827 (1.216)		−0.026 (0.259)	
Exposure to ≥ 1 Interventions	0.955** (0.354)	0.954** (0.354)	0.327 (0.203)	0.326 (0.203)	0.414 (0.229)	0.414 (0.228)	0.608** (0.179)	0.608** (0.178)	0.200** 0.069	0.200** 0.069	0.292 (0.246)	0.287 (0.245)	0.302 (0.284)	0.287 (0.245)	0.133** (0.060)	0.133** (0.060)
Age of caregiver	0.056** (0.017)	0.056** (0.017)	0.001 (0.010)	0.000 (0.010)	−0.001 (0.011)	−0.002 (0.012)	0.O100.008	0.O100.008	−0.004(0.003)	−0.004 (0.003)	0.022** (0.011)	0.021** (0.011)	0.025** (0.014)	0.021 (0.011)	0.0010.003	0.001 (0.003)
Household size	−0.012 (0.090)	−0.012 (0.090)	−0.079 (0.128)	−0.079 (0.052)	−0.069 (0.058)	−0.069** (0.058)	−0.011 (0.044)	−0.010 (0.044)	0.032** (0.017)	0.032** (0.017)	−0.122** (0.062)	−0.119 (0.062)	−0.059 (0.072)	−0.119 (0.062)	−0.006 (0.015)	−0.007 (0.015)
Education of caregiver																
No Schooling	Ref	Ref	Ref	Ref	Ref	Ref	Ref	Ref	Ref	Ref	Ref	Ref	Ref	Ref	Ref	Ref
Primary School completed	3.114** (0.666)	3.113** (0.666)	0.861** (0.422)	0.861** (0.422)	0.942** (0.475)	0.944** (0.474)	1.068** (0.392)	1.067** (0.391)	0.241** (0.144)	0.241** (0.144)	−0.417 (0.462)	−0.408 (0.462)	0.231 (0.591)	−0.408 (0.462)	−0.018 (0.125)	−0.020 (0.125)
Above Primary School Completed	5.619 (0.720)	5.619 (0.720)	1.196** (0.450)	1.197** (0.451)	1.288** (0.507)	1.293** (0.504)	0.868 (0.409)	0.867 (0.408)	0.341** (0.153)	0.341** (0.153)	−0.179 (0.500)	−1.164 (0.499)	0.887 (0.631)	−0.164 (0.499)	0.089 (0.134)	0.085 (0.133)
Market participation																
No	Ref	Ref	Ref	Ref	Ref	Ref	Ref	Ref	Ref	Ref	Ref	Ref	Ref	Ref	Ref	Ref
Yes	0.898** (0.351)	0.898** (0.351)	1.197** (0.221)	1.197** (0.221)	0.883** (0.415)	0.798** (0.244)	0.466**0.171	0.466**0.170	−0.079 (0.075)	−0.079 (0.075)	1.318 (0.244)	1.286** (0.237)	1.014** (0.310)	1.286** (0.237)	−0.018 (0.117)	0.025 (0.064)
Involved in informal business																
No	Ref	Ref	Ref	Ref	Ref	Ref	Ref	Ref	Ref	Ref	Ref	Ref	Ref	Ref	Ref	Ref
yes	−0.493 (0.434)	−0.493 (0.434)	−0.028 (0.282)	−0.028 (0.282)	−0.049 (0.318)	−0.049 (0.317)	0.102 (0.203)	0.101 (0.202)	0.041 (0.096)	0.041 (0.096)	0.858 (0.301)	0.846 (0.300)	0.218 (0.396)	0.846** (0.300)	0.067 (0.084)	0.067 (0.084)
Salaried employment within the past 1 year																
No	Ref	Ref	Ref	Ref	Ref	Ref	Ref	Ref	Ref	Ref	Ref	Ref	Ref	Ref	Ref	Ref
Yes	−0.625 (0.048)	−0.625 (0.408)	0.360 (0.253)	0.360 (0.253)	0.494** (0.285)	0.491** (0.283)	−0.255 (0.198)	−0.254 (0.198)	0.012 (0.086)	0.012 (0.086)	0.748 (0.283)	0.741** (0.283)	0.595 (0.354)	0.741** (0.283)	0.056 (0.15)	0.059 (0.075)
Agriculture is principal activity																
No	Ref	Ref	Ref	Ref	Ref	Ref	Ref	Ref	Ref	Ref	Ref	Ref	Ref	Ref	Ref	Ref
Yes	0.133*8 (0.075)	0.133 (0.092)	0.638** (0.225)	0.682 (0.225)	0.520 (0.253)	0.521 (0.253)	−0.644 (0.856)	−0.644 (0.856)	−0.613 (0.633)	−0.613 (0.633)	−0.613 (0.611)	−0.644 (1.054)	−0.644 (1.110)	0.643 (0.261)	0.073 (0.067)	0.074 (0.067)
Access to off- farm income																
No	Ref	Ref	Ref	Ref	Ref	Ref	Ref	Ref	Ref	Ref	Ref	Ref	Ref	Ref	Ref	Ref
Yes	0.011 (0.564)	0.011 (0.564)	0.671 (1.197)	0.671 (0.369)	0.833** (0.415)	0.881** (0.413)	0.0720.287	0.0710.286	−0.127 (0.125)	−0.127 (0.125)	0.112 (0.392)	0.123 (0.391)	0.280 (0.516)	0.123 (0.391)	0.062 (0.109)	0.064 (0.109)
Distance to source of water in dry season	−0.634 (0.376)	−0.007** (0.003)	−0.002 (0.002)	−0.002 (0.002)	−0.003 (0.002)	−0.004** (0.002)	−0.004** (0.001)	−0.004** (0.001)	−0.001 (0.001)	−0.000 (0.000)	−0.007** (0.002)	−0.007 (0.002)	−0.001 (0.003)	−0.001** (0.002)	−0.001 (0.001)	−0.001 (0.001)
Currently grow sweet potato																
No	Ref	Ref	Ref	Ref	Ref	Ref	Ref	Ref	Ref	Ref	Ref	Ref	Ref	Ref	Ref	Ref
Yes	0.577 (0.336)	0.577** (0.336)	0.153 (0.207)	0.154 (0.208)	0.127 (0.239)	0.125(0.232)	0.500** (0.164)	0.500** (0.164)	0.0210.070	0.0210.070	0.794** (0.234)	0.794** (0.233)	0.436 (0.291)	0.794** (0.233)	0.012 (0.062)	0.014 (0.061)
Wealth index																
Lowest	Ref	Ref	Ref	Ref	Ref	Ref	Ref	Ref	Ref	Ref	Ref	Ref	Ref	Ref	Ref	Ref
Medium	0.650 (0.395)	0.650 (0.101)	0.167 (0.248)	0.167 (0.249)	0.404 (0.279)	0.406 (0.278)	0.197 (0.192)	0.197 (0.192)	−0.021 (0.084)	−0.021 (0.084)	−0.286 (0.297)	−0.272 (0.273)	−0.268 (0.348)	−0.272 (0.273)	−0.024 (0.04)	−0.026 (0.073)
Highest	0.440 (0.424)	0.440 (0.424)	0.391 (0.252)	0.391 (0.252)	0.576** (0.283)	0.580** (0.279)	0.038 (0.205)	0.038 (0.205)	0.036 (0.085)	0.036 (0.085)	−0.492** (0.295)	−0.480 (0.294)	−0.208 (0.352)	−0.480 (0.294)	0.026 (0.075)	0.023 (0.074)

**, *** represents *p* < 0.05, β represents coefficient while SE indicates standard error.

## Discussion

Humanitarian programming in the arid and semi-arid lands (ASALs) targeted at addressing recurrent shocks has mostly taken the form of emergency aid with a main goal of providing immediate lifesaving solutions to the most vulnerable segments of the population (44). Such programs are mostly focused on short-term emergency food support with little emphasis on resilient building of the affected population for more sustainable or long-term impact (45). Recently, humanitarian agencies such as the WFP (46) have moved toward more sustainable humanitarian interventions including climate-smart NSA (47). Main emphasis of these NSA interventions is placed on production of diverse nutrient dense crops coupled with behavior change communication aimed at enhancing the consumption of such nutritious foods to improve food and nutrition security (10, 48). This current study assessed the effect of participation in a climate-smart NSA intervention program on caregiver knowledge and practices in ASAL regions of Kenya. The interventions included accessing planting materials of VA-rich OFSP for cultivation, attending nutrition group meetings and receiving information on nutrition social behavior change communication (SBCC), and receiving innovative feeding toolkits (healthy baby toolkit/HBT) and information on how to utilize these to improve infant and maternal feeding in the household.

Households that participated in at least one of the study interventions had significantly higher caregiver nutrition and VA knowledge scores, young child minimum meal frequency (MMF) and minimum acceptable diet (MAD) compared to non-participating households. We observed similar results from our previous studies in western Kenya (38) and in rural Tanzania (17). In the Kenya study, women who attended at least one antenatal care (ANC) clinic had significantly better health and nutrition knowledge score compared to non-attending women; while for the Tanzania study, participation in nutrition group meetings was significantly associated with the health and childcare knowledge score (HKS), household (HDD) and young child (MDD-C) minimum diversity scores, and household and young child VA intake. In this current study, we found that participating in at least one of the interventions was associated with improved diet quality (MMF and MAD) among children 6–23 months of age. These results were similar to those observed in rural Bangladesh among women participating in a community-based nutrition education program; infants of women who were involved in the intervention had significantly increased MAD at 9 months of age compared to non-participants (49).

We examined the effect of each of the individual interventions on the caregiver KP and found that, participation in nutrition education sessions improved caregiver knowledge in nutrition and VA and subsequently, caregiver practices in improved young child MMF and MAD. This suggests that nutrition and health education that was provided in the program’s nutrition group meetings potentially improved nutrition and infant care knowledge and improved practices, culminating in improved MMF and MAD for children of these caregivers. Poor food choices coupled with lack of knowledge about the importance of food group diversity for the health and growth of young children, as well as lack of availability of or decreased accessibility to certain foods can potentially restrict the inclusion of micronutrient-rich foods in the diets of young children (50–52). In this study, receiving the innovative feeding toolkits and information on how to utilize these for improved maternal and infant feeding practices resulted in improved caregiver knowledge (in nutrition and VA). Thus, the toolkit was associated with higher caregiver knowledge, women’s and children’s dietary diversity, infant VA intake, MMF and MAD among this sample. Similar findings were observed in India (25), Kenya (20), Malawi (26) and Ethiopia (53) where the toolkit was found to be easy to use by families, highly acceptable and with potential to have substantial impacts on critical infant and young child diets.

In our study, access to biofortified VA-rich OFSP planting materials was significantly associated with minimum diet diversity for both women (MDD-W) and children (MDD-C), as well as infant MAD. The access to OFSP planting materials was accompanied with provision of extension services as well as knowledge on good agronomic practices (GAP) contained in information, education, and communication (IEC) materials (e.g., brochures) to participating households (54) found that the provision of GAP alongside agriculture inputs was directly associated with increased yields and hence, household food availability. This has the potential to improve both household and young child diet diversity as was observed in our study. Findings from our study parallels those from previous studies that introduced OFSP with strong community-level nutrition education in Mozambique (13, 55) and Uganda (56) which showed a high uptake of OFSP cultivation, and consequently resulted in improved intakes of VA rich foods, culminating in reduction in young child VAD. Similar findings were observed in our previous study in western Kenya, where pregnant and lactating women participating in an intervention that promoted OFSP through health services, achieved improved diet diversity, nutrition knowledge, VA intakes, and maternal retinol binding protein – an indicator of VA status - compared to controls (15). Our study also reported an improved MDD-W and MDD-C among households that participated in market activities. This indicates that women who were involved in the income-generating activities in our study might potentially have had the means and autonomy to decide on the acquisition of a quality diet for the family and children in the study’s intervention sites. Households that were involved in market activities might have been economically stable and thus, have the means to access nutritious foods for household consumption (17). The finding is however, in contrast with previous evidence that caregiver involvement in employment, such as in market activities, is associated with maternal “double burden” of increased demands for labor and economic activity to the detriment of adequate childcare responsibilities (57).

In conclusion, this study showed that in fragile environments, household participation in climate smart nutrition sensitive agriculture interventions (comprising of nutrition training, use of infant feeding toolkit and / or access to orange-fleshed sweetpotato (OFSP) vines for cultivation) was significantly associated with improved caregiver nutrition and VA knowledge scores, young child MMF and MAD compared to non-participating households. Independently, households that participated in any one of the three interventions separately benefited from improved outcomes such as caregiver VA knowledge, infant MMF, as well as caregiver (MDD-W) and infant (MDD-C) diet quality. The findings from this study indicate the need to integrate NSA interventions in humanitarian settings to improve nutrition among women and young children. These NSA interventions can potentially build resilience among the population in these fragile environments to withstand various shocks.

## Data Availability

The original contributions presented in the study are included in the article/supplementary material, further inquiries can be directed to the corresponding author/s.

## Glossary

ANC: Antenatal clinic
ASAL: Arid and semi-arid lands
CAPI: Computer-assisted personal interview
CHVs: Community health volunteers
CIP: International potato center
DDBIO: Development and delivery of biofortified foods at scale
DDS: Dietary diversity score
DVMs: Decentralized vine multipliers
GAP: Good agronomic practices
HBT: Healthy baby toolkit
HFIAS: Household food insecurity assessment scale
HH: Household
HKI: Helen Keller international
IEC: Information, education, and communication
ILRI-IREC: International Livestock Research Institute’s Institutional Research Ethic Committee
IPs: Implementing partners
IPWRA: Inverse probability weighting regression adjustment
KP: Knowledge and practices
LMICs: Low- and middle-income countries
MAD: Minimum acceptable diet
MDD-C: Minimum dietary diversity for children
MDD-W: Minimum dietary diversity for women
MMF: Minimum meal frequency
NACOSTI: National Commission for Science, Technology and Innovation
NKS: Nutrition knowledge score
NSA: Nutrition-sensitive agriculture
OFSP: Orange fleshed sweet potato
PPS: Probability proportional to size
SBCC: Social behavior change communication
VA: Vitamin A
VAD: Vitamin A deficiency
VAKS: Vitamin A knowledge score
WAEOs: Ward agriculture extension officers
WFP: World Food Program of the United Nations
WRA: women of reproductive age
